# Trends and threshold exceedances analysis of airborne pollen concentrations in Metropolitan Santiago Chile

**DOI:** 10.1371/journal.pone.0123077

**Published:** 2015-05-06

**Authors:** Richard Toro A., Alicia Córdova J., Mauricio Canales, Raul G. E. Morales S., Pedro Mardones P., Manuel A. Leiva G.

**Affiliations:** 1 Centro de Ciencias Ambientales and Departamento de Química, Facultad de Ciencias, Universidad de Chile, Casilla 653, Santiago, Chile; 2 Clínica de Enfermedades Respiratorias y Alérgicas Miguel Servet, Almirante Pastene N° 150–118, Providencia, Santiago, Chile; 3 Fundación de Aerobiología Medio Ambiente y Salud, Pérez Valenzuela 1572, Of 404, Santiago, Chile; Georgia State University, UNITED STATES

## Abstract

Pollen is one of the primary causes of allergic rhinoconjunctivitis in urban centers. In the present study, the concentrations of 39 different pollens in the Santiago de Chile metropolitan area over the period 2009–2013 are characterized. The pollen was monitored daily using Burkard volumetric equipment. The contribution of each type of pollen and the corresponding time trends are evaluated. The concentrations of the pollens are compared with the established threshold levels for the protection of human health. The results show that the total amount of pollen grains originating from trees, grasses, weeds and indeterminate sources throughout the period of the study was 258,496 grains m-3, with an annual average of 51,699 ± 3,906 grains m-3 year-1. The primary source of pollen is Platanus orientalis, which produces 61.8% of the analyzed pollen. Grass pollen is the third primary component of the analyzed pollen, with a contribution of 5.82%. Among the weeds, the presence of Urticacea (3.74%) is remarkable. The pollination pattern of the trees is monophasic, and the grasses have a biphasic pattern. The trends indicate that the total pollen and tree pollen do not present a time trend that is statistically significant throughout the period of the study, whereas the grass pollen and weed pollen concentrations in the environment present a statistically significant decreasing trend. The cause of this decrease is unclear. The pollen load has doubled over the past decade. When the observed concentrations of the pollens were compared with the corresponding threshold levels, the results indicated that over the period of the study, the pollen concentrations were at moderate, high and very high levels for an average of 293 days per year. Systematic counts of the pollen grains are an essential method for diagnosing and treating patients with pollinosis and for developing forestation and urban planning strategies.

## Introduction

Pollen is one of the most important triggers of allergic inflammation in the nasal, conjunctival and/or bronchial mucosa [1–3]. The majority of patients with allergic diseases present clinical symptoms in the respiratory tract [1,3]. In the nasal mucosa, the inflammation produces a set of symptoms that typically include sneezing, nasal itch, congestion and nasal discharge and blockage [3]. Typically, other tissues are also affected, which may result in conjunctivitis or an itchy throat [4]. In addition, the entire respiratory tract may be affected, which increases the seriousness of the symptoms and complicates the disease management [5–8].

Two primary events are required for an allergic reaction to occur. First, a process of primary sensitization occurs in which a non-allergic person, after repeated exposure to certain pollens, becomes allergic, *i*.*e*., the person experiences a series of immunological changes that lead to the production of specific antibodies of the immunoglobulin E type that are then attached to specialized cells called mast cells [9,10]. Second, after an additional exposure to the same type of pollen, these activated mast cells release a variety of substances into the surrounding tissues and blood stream that cause the clinical symptoms of allergic rhinoconjunctivitis and, occasionally, bronchial asthma [11,12]; these processes are called pollinosis. Significant costs to the economy related to allergic rhinitis are 1) loss of productivity and 2) days away from work.

The effect that pollen has on human health is difficult to define and quantify [13]. The capacity to generate symptoms of an allergy after exposure to pollen depends on certain factors, such as the species, genus and family (*i*.*e*., the taxa) along with its concentration in the environment, length of the period over which it is released to the atmosphere and the expression of antigenic determinants in each grain of pollen [13,14]. Additional factors include the exposure time, meteorology and the interaction between the pollen and the particulate matter and/or other pollutants that may also be present in the environment [15–17]. In addition, the degree of susceptibility of the population is also involved, *i*.*e*., the percentage of people who are sensitized to certain pollens present in the environment [18,19]. Thus, information on the presence and concentration of airborne pollens aids in the diagnosis and prevention of pollinosis, contributes to the planning of clinical studies and allows for the selection of an optimal time for prophylactic treatment [20–22]. Systematic measurements of the airborne pollen concentration have been conducted in different parts of the world [23–30]; however, in the southern hemisphere, these types of measurements have been rare.

In Chile, seminal studies conducted in the Santiago de Chile Metropolitan Area (SChMA) during the 1970s [23,31–34] have provided the first qualitative information on the different types of pollen present in the urban environment of the SChMA. In the study by Hoffman et al. in 1976 [32], the abundance of pollen from *Poaceae* and *Populus* along with the rare presence of pollen from *Platanus* was noticeable. During the 1990s, systematic counts of airborne pollen grains performed in the SChMA over three years (1993–1996) using volumetric methods were reported, and they showed that airborne pollen primarily arises from certain taxa, such as *Platanus*, *Acer*, Poaceae, *Morus*, *Plantago*, *Cupressus*, Chenopodiaceae, *Urticaceae* and *Oleaceae* (arranged by the size of their relative pollen contribution, from greatest to smallest). However, to our knowledge, there have been no additional reports on the subject in the literature. In the case of the SChMA, because of its rapid growth and territorial expansion [35], it has been particularly important to characterize, update and systemize the available information on the types and corresponding concentrations of the pollen present in its urban environment. Recent studies conducted in the cities of Temuco [36,37], Talca [38] and Valparaíso [39] have also been notable because they have provided knowledge regarding the types of airborne pollen present in these cities and their corresponding concentrations.

The present study describes the results of research performed in the pollen-monitoring station located in the Providencia County of the SChMA. The airborne pollen concentrations and their corresponding long-term trends and seasonal variations are analyzed from the pollen grain counts conducted from 2009 and 2013. Thirty-nine taxa are identified that correspond to 28 taxa of trees, 10 taxa of weeds or herbs and one taxon of grass. Pollens from indeterminate or non-identified sources are also included in the analysis. A comparison of the results with previous data is performed to analyze the evolution of the concentrations or the types of airborne pollen found in the SChMA. Finally, possible strategies for controlling plants that produce and release allergenic pollen are proposed.

## Materials and Methods

### Study area

**Fig 1:**
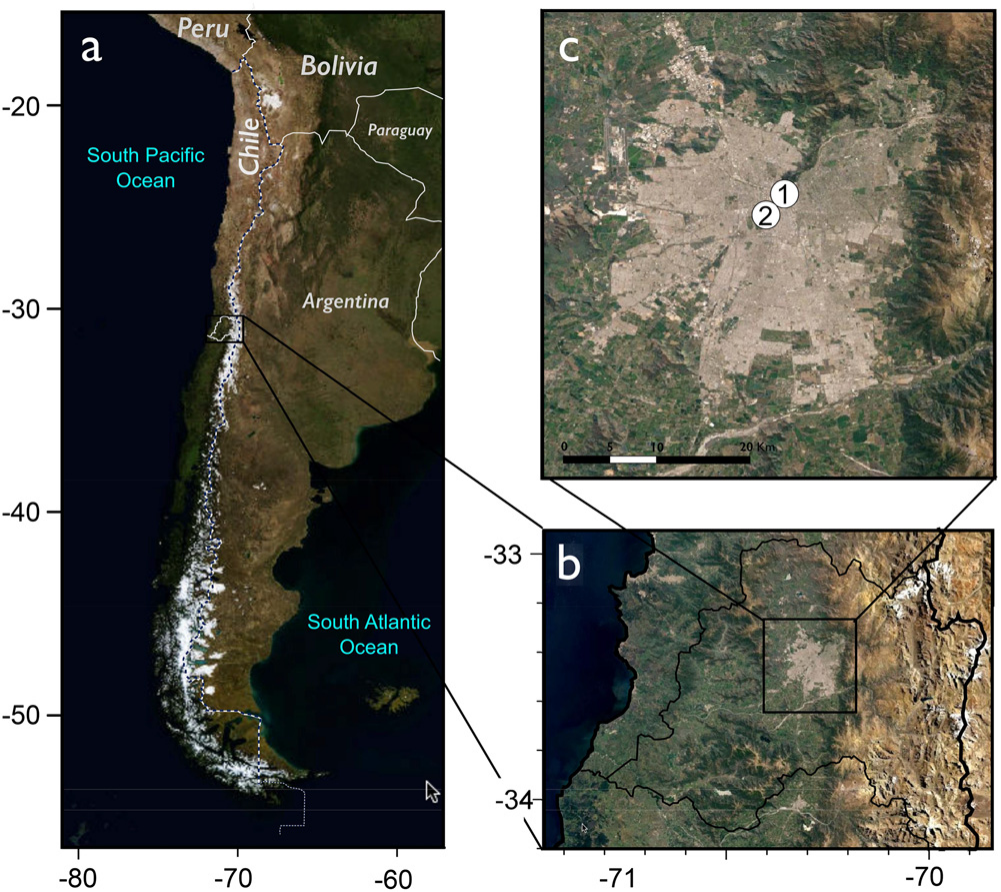
Location of the study area. (1) Pollen-sampling location. (2) Location of the meteorological monitoring station. Map sources: USGS The National Map Viewer (http://viewer.nationalmap.gov) and USGS Global Web—Enable LANSAT data (http://globalweld.cr.usgs.gov).

The Santiago de Chile Metropolitan Area (SChMA; 33°27´S, 70°38´W, Fig 1) is the capital of the Republic of Chile and is the financial, industrial and political center of the country [40]. The city is located in a valley that is flanked to the east by the Andes chain (with an average altitude of 4,500 m), to the west by the Chilean Coastal Range (with an average altitude of 1,500 m) and to the north and south by two ranges extending from east to west. The population in the SChMA has doubled since 1980 to approximately 6.5 million, representing over 40% of the country’s inhabitants [40]. The urban area of SChMA is divided into 34 communities. Approximately one million people reside and work in the community of Providencia (the location of the sampled pollen). The community is characterized by a significant presence of businesses, with several offices and residential areas. The climate is Mediterranean, and the temperature oscillates from—2°C to 35°C, with an average of approximately 14°C [41].

### Characterization of the local vegetation

The vegetation in the city of the SChMA is characteristically composed of sclerophyll forest and arborescent thorny bushes; thus, it is dominated by perennials [42]. In the green spaces, exotic, introduced and ornamental species are dominant over the other taxa, such as *Cupressus sempervirens*, *Pinaceae*, *Taxodiaceae*, *Gymnospermae*, *Platanus orientalis*, *Acer negundo*, *Populus alba* and *Ulmus pumila*, among others. These species pose an additional risk for causing and exacerbating allergy symptoms. Planting these types of ornamental vegetation may result in additional exposure to allergic components that provide new sources of aeroallergens [43–46]. Introduced weeds have also been observed, of which the Gramineae and weeds from the taxa *Asteraceae*, *Ambrosia elatior* and *Chenopodium album* are dominant. Species from the genera *Salix* and *Populus* have also been found.

### Airborne pollen sampling

The airborne pollen concentration and composition were analyzed from January 1, 2009, to December 31, 2013. A Burkard volumetric sampler (Burkard Seven Day Spores-Trap, Burkard Manufacturing Co. Ltd., Hertfordshire, UK) was used to collect the airborne pollen grains and to determine their concentration [47]. The equipment was placed on the roof of the Clínica Miguel Servet building (33°25'38"S, 70°37'13"W, 600 m.a.s.l.) in adherence with the guidelines established by the World Allergy Organization [48]. The sampling location is 3 km (1.86 miles) NEE from the center of the city in Providencia County. The pollen sampler operates by the intake of air at a flow of 10 L min^–1^ (corresponding to the average human respiratory flow) and the application of force against the air to collide with tape covered with Melinex, thus trapping the airborne particles and pollen grains. The tape is located inside of an aspiration chamber and covers the surface of a rotatory drum that completes one turn per week (i.e., the drum completes a rotation every seven days at a speed of 2 mm hr^-1^). This strip is removed every week and cut into 48-mm-long segments, each of which corresponds to one day of sampling. To maintain continuity in monitoring the pollen over the required course of time, the tape must be changed once per week.

The pollen samples were processed and analyzed using a longitudinal “read” of the tapes in which each tape was set on a plaque and the pollen grains that adhered to it were counted on a daily basis using 400 x magnification microscope optics in accordance with the procedures and formulas standardized for this process [48]. The results are expressed in the units of grain m^-3^ day^-1^.

The identified pollens from the trees, weeds and grasses are listed in Table 1. The analysis methodology has been granted a national and international certification by the Chilean Society of Allergy and Immunology [49] and the American Academy of Allergy Asthma and Immunology [50], respectively.

**Table 1:** Pollen taxa analyzed in the Santiago de Chile Metropolitan Area in Chile.

Common Name	Scientific name (Family)
Trees	
Abedul	*Betula verrucosa* (Betulaceae)
Acacia p	*Robinia pseudoacacia* (Fabaceae)
Acacia d	*Acacia dealbata* (Fabaceae)
Ailanthus	*Ailanthus altissima* (Simaroubaceae)
Acer	*Acer negundo* (Aceraceae)
Chestnut	*Castanea sativa* (Fagaceae)
Cypress	*Cupressus sempervirens* (Cupressaceae)
Espadaña, totora	*Typha angustifolia* (Typhaceae)
Eucalyptus	*Eucalyptus globulus* (Myrtaceae)
Fresno	*Fraxinus excelsior* (Oleaceae)
Hazelnut	*Spanish avellano* (Betulaceae)
Jacaranda	*Jacaranda mimosifolia* (Bignoniaceae)
Ligustrum	*Ligustrum lucidum* (Oleaceae)
Liquidambar	*Liquidambar styraciflua* (Altingiaceae)
Morus	*Morus alba* (Morus)
Nogal	*Juglans regia* (Juglandaceae)
Oak	*Quercus robur* (Fagaceae)
Olive	*Olea europaea* (Oleaceae)
Palm	*Palmae* (Arecaceae)
Patagua	*Crinodendron patagua* (Elaeocarpaceae)
Pepper tree	*Schinus Molle* (Anacardiaceae)
Pinus	*Pinus spp*. (Pinaceae)
Platanus hispánica	*Platanus orientalis* (Platanaceae)
Poplar	*Populus alba* (Salicaceae)
Tilia	*Tilia americana* (Tiliaceae)
Ulmus	*Ulmus pumila* (Ulmaceae)
Willow	*Salix babylonica* (Salicaceae)
**Weeds**	
Acedera	*Rumex acetocela* (Eriophyidae)
Ambrosia	*Ambrosia elatior* (Asteraceae)
Asteraceae	*Compositae spp*. (Compositae)
Carex	*Carex hirta* (Cyperaceae)
Chenopodium	*Chenopodium album* (Chenopodiaceae)
Colliguaja	*Colliguaja odorifera* (Euphorbiaceae)
Urticacea	*Parietaria* (Urticaceae)
Plantago	*Plantago lanceolata* (Plantaginaceae)
Umbeliferas	*Umbeliferas spp*. (Apiaceae)
Yuyos	*Brassica napus and campestris* (Brassicaceae)
**Grasses**	
Grasses	*Poaceae* or *Gramineae* (Poaceae)

### Meteorological data

The meteorological data were obtained from the National Air Quality Information System [51], which is currently operated by the Chilean Ministry of the Environment. The data were recorded at an automatic station located 5.6 km (3.48 miles) south of the pollen sampling site (in the Parque O´Higgins station; 33°27’40”S, 70°39’29”W). The data include the average daily temperature, the relative humidity and the daily predominant direction and speed of the wind.

### Statistical analysis

A descriptive statistical analysis of the pollen database based on a daily concentration was performed using MS Excel 2011 for Mac (Microsoft, Redmond, WA, US) and the statistical programming language R [52] using the OpenAir package [53,54], which was executed under the control of the RStudio Desktop version 0.98.953 software, an integrated development environment for R [55]. The programming language R and the OpenAir package are open-source software and are available online.

The time trends were estimated using the Theil-Sen approach [56,57] in the OpenAir package. The deseason option was applied to remove the seasonal variability from the time series [53]. The Theil-Sen approach provides consistency between the p-value and the uncertainty intervals in the slope. Our study shows that the Mann-Kendall test for the slope and its associated p-value lead to results similar to the Theil-Sen estimates. A positive slope indicates an increasing trend, and a negative slope indicates a decreasing trend. A significant trend was identified when the confidence intervals at the 95% confidence level did not include a zero slope.

For the bivariate polar plot of the weighted mean of the total concentration of the airborne pollen (*i*.*e*., the product of the concentration and the frequency of occurrence), which also highlights the speed and direction of the wind that dominates the overall mean, the polar plot option in OpenAir was used [53].

## Results and Discussion

### Relative contributions to the total concentration of airborne pollen

**Fig 2:**
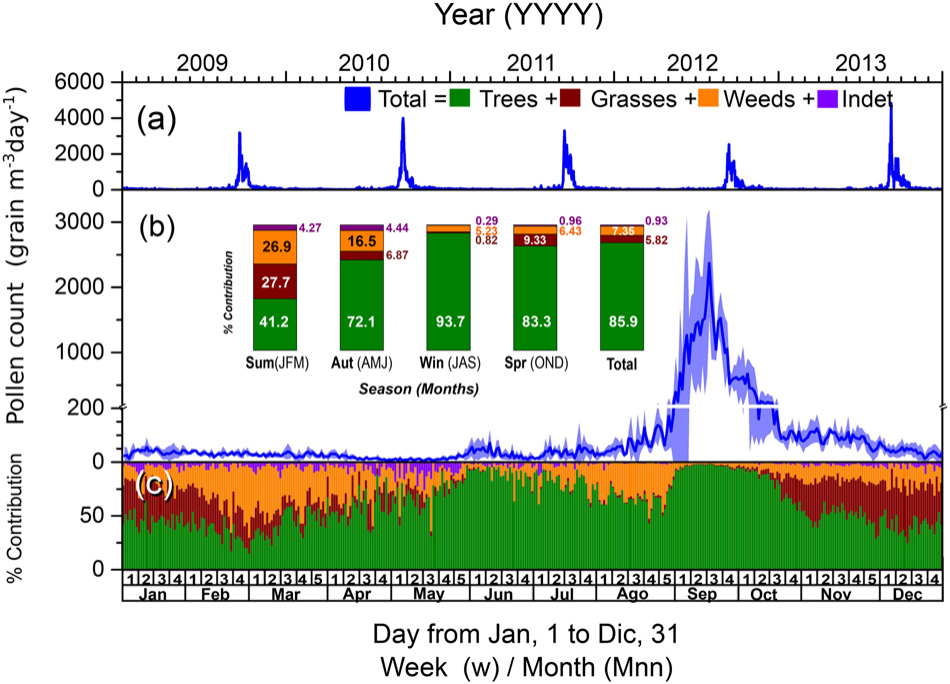
Time series of the total daily concentration of the airborne pollen grains (equal to the sum of the pollen grains from trees, grasses, weeds and indeterminate sources in grains m–3 day–1) collected over the study period (from the January 1, 2009, to December 31, 2013). (a) Time series per year (top axis). (b) Average daily counts of the airborne pollen grains with their corresponding standard deviations for the five-year (bottom axis) and annual relative contribution of each group of pollens by the season, and (c) the relative contribution of each group per annual season (top axis). Average daily relative contribution of tree, grass, weed and indeterminate source pollens to the total amount of airborne pollen (bottom axis).

Fig 2a presents the time series of the total daily concentration of the airborne pollen grains (m^-3^ day^-1^) collected over the five-year study. Fig 2b shows the average daily counts for the five-year study and their corresponding standard deviations and depicts the seasonal variation by pollen group (the analyzed pollen grains were classified into the following four groups: trees, grasses, weeds and indeterminate sources). Fig 2c shows the average of the daily relative contribution to the total amount of pollen in each group over one year. Table 2 lists the total amount of airborne pollen grains collected each year and for each group, along with their corresponding daily maximums.

**Table 2:** Total counts of the airborne pollen grains and counts of the airborne pollen grains per group (tree, grass, weed and indeterminate sources) per year and for the entire period of the study (expressed as grains m^–3^ year^–1^) and their corresponding average and standard deviation (SD) per year (expressed as grains m^–3^ year^–1^).

	Total	Trees	Grass	Weeds	Indet
**Year**	**Pollen grains**				
2009	48,659	39,973	3,469	4,516	701
2010	54,517	46,713	3,267	4,000	537
2011	54,247	47,465	2,995	3,373	414
2012	46,374	39,293	2,997	3,697	387
2013	54,699	48,613	2,320	3,403	363
**Avg**	**51,699**	**44,411**	**3,010**	**3,798**	**480**
**SD**	**3,906**	**4,421**	**434**	**475**	**140**
**Total**	**258,496**	**222,057**	**15,048**	**18,989**	**2,402**
**Year**	**Daily Maximum pollen grains**				
2009	3,194	3,194	74	67	14
2010	4,004	4,004	58	66	12
2011	3,304	3,304	48	53	7.0
2012	2,539	2,539	98	52	10
2013	4,830	4,830	41	88	12
**Avg**	**3,574**	**3,522**	**64**	**65**	**65**
**SD**	**873**	**867**	**23**	**15**	**15**

The table also shows the corresponding maximum daily counts observed in each year of the study (expressed as grains m^–3^ year^–1^) and the seasonal contribution of the different pollens.

The total amount of pollen grains originating from trees, grasses, weeds and indeterminate sources that were collected over the study period (from 2009 to 2013) was 258,496, with an annual average of 51,699 ± 3,906 grains m^–3^ year^–1^ (Table 1). The relative contributions observed for the groups of indeterminate source: grasses: weeds: trees were 0.2: 1.0: 1.3: 14.8, respectively. The maximum daily total count for each year was over the range of 54,699 to 46,374 grains m^–3^ day^–1^. The lowest maximum value was registered in 2012, and the highest value was recorded in 2013 (Table 1 and Fig 2a). Generally, the highest concentration of airborne pollen was observed at the end of September and in early October (Fig 2b). The composition of the analyzed pollen samples showed that tree pollen (Table 1, Fig 2b and 2c) dominated over weed, grass and indeterminate source pollens. This result was verified by the fact that tree pollen grains represented 86% of the total airborne pollen grains collected over the study period, whereas pollen grains from grasses and weeds represented 5.82% and 7.35%, respectively. However, the pollen grains of an indeterminate source represented 0.93% of the total airborne pollen grains collected. There was a strong lineal correlation (R^2^ value of 0.997; and a slope of 0.962 ± 0.003) between the daily concentrations of the tree pollen and amounts of the total pollen grains altogether, which confirms the high contribution of tree pollen to the total amount of airborne pollen.

For the seasonal variation observed in the pollen grain count (Fig 2b), a greater proportion of total pollen grains was observed during the winter (52.4%) and spring (38.1%), whereas the lowest proportions of total pollens were observed during the summer (5.70%) and autumn (3.76%). In the case of the tree pollens, the greatest contribution of the total pollen grains per season was observed during the autumn, winter and spring, with contributions of over 70%. A higher proportion was observed for the grass pollens during the summer, reaching approximately 28%, followed by the spring and autumn, with a contribution of approximately 8%. For the grass pollens, the largest proportion was observed during the summer and autumn, with contributions over the range of 16–27% of the pollen grains. These seasonal patterns are regulated by various climatic factors and by flowering periods. In the future, climate change will affect the allergenic pollens, thus increasing the level of chronic illnesses. The possible changes in the temperature, via cold events or heat events, are factors that will require increasing attention in the next few decades.

**Fig 3:**
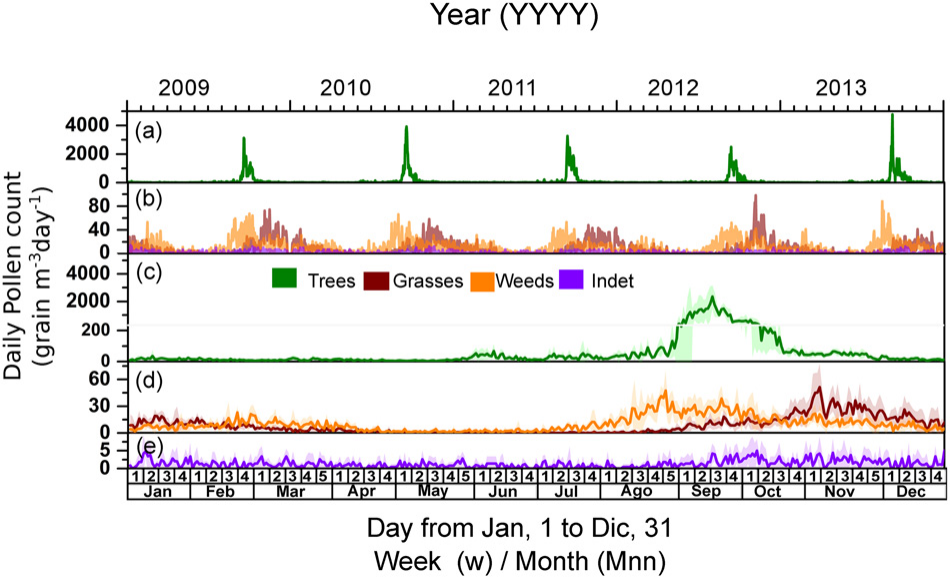
Time series of the daily concentration of the airborne pollen grains (expressed as grains m^–3^ day^–1^) from trees, grasses, weeds and indeterminate sources over the period of the study (from January 1, 2009, to December 31, 2013). (a) Time series of the tree pollen concentration (top axis); (b) time series of the concentrations of pollen from grasses, weeds and indeterminate sources (top axis); (c) average tree pollen grain counts per day and the corresponding standard deviations; (d) average grass-pollen and weed-pollen grain counts per day and their corresponding standard deviations; and (e) average counts of the pollen grains from indeterminate sources per day and their corresponding standard deviations.

Fig 3 shows the time series and the average (along with the corresponding standard deviations) of the daily concentrations of the airborne pollen over the five-year study estimated for each group. The expected sequential pattern of the pollen release is observed, with trees starting to pollinate during the spring once winter is finished (Fig 3a–3c) and peaking in September; grasses started to pollinate between the spring and early summer, peaking between the end of October and the beginning of December (Fig 3b–3e); and weeds started to pollinate between the summer and early autumn (Fig 3b–3e).

### Tree pollen

**Fig 4:**
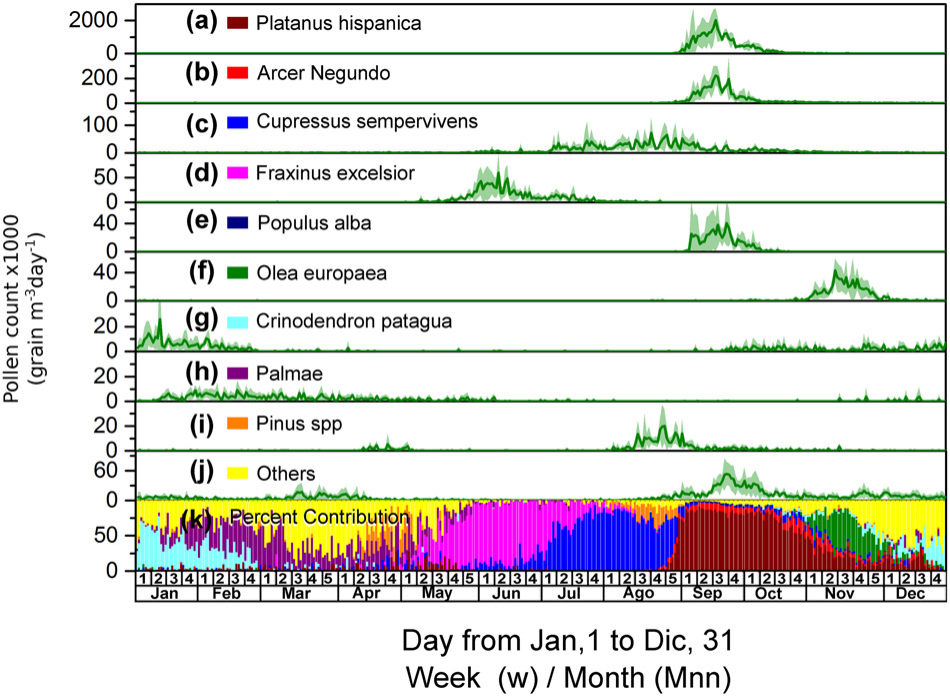
Average daily counts and relative contribution of the tree airborne pollen. (a to j) Average daily counts of the pollen grains from the most representative tree taxa and their corresponding standard deviations (expressed as grains m^–3^ day^–1^). (k) Relative contribution of each tree taxon to the daily counts of the tree pollen grains.

The records of airborne pollen from trees indicate that among the 28 identified taxa (Fig 4a–4i), there are nine predominant taxa, as follows: *Platanus orientalis* (with an annual average pollen grain concentration of 31,951 ± 3,820 grains m^–3^ year^–1^, which represents 72.0% of the tree pollen), *Acer negundo* (3,394 ± 375 grains m^–3^ year^–1^; 7.64%), *Cupressus sempervirens* (2,572 ± 231 grains m^–3^ year^–1^; 5.79%), *Fraxinus excelsior* (1,162 ± 124 grains m^–3^ year^–1^; 2.62%), *Populus alba* (648 ± 79 grains m^–3^ year^–1^; 1.40%), *Olea europaea* (621 ± 116 grains m^–3^ year^–1^; 1.46%), *Crinodendron patagua* (545 ± 66 grains m^–3^ year^–1^; 1.23%), Palmae (477 ± 44 grains m^–3^ year^–1^; 1.07%) and *Pinus spp*. (419 ± 48 grains m^–3^ year^–1^; 0.94%). The remaining taxa contribute 2616 ± 131 grains m^–3^ year^–1^, representing 5.9% of the tree pollen. Fig 4k indicates the daily relative contribution to the annual profile of each of the predominant taxa.

The minority tree pollen taxa included in the category “others” (Fig 4j) were as follows: *Juglans regia* (406 ± 51 grains m^–3^ year^–1^; 0.91%), *Corylus* (396 ± 66 grains m^–3^ year^–1^; 0.89%), *Schinus molle* (292 ± 40 grains m^–3^ year^–1^; 0.66%), *U*. *pumila* (268 ± 41 grains m^–3^ year^–1^; 0.60%), *Eucalyptus globulus* (196 ± 22 grains m^–3^ year^–1^; 0.44%), *Morus alba* (188 ± 48 grains m^–3^ year^–1^; 0.42%), *Castanea sativa* (166 ± 28 grains m^–3^ year^–1^; 0.37%), *Liquidambar styraciflua* (139 ± 25 grains m^–3^ year^–1^; 0.31%), *Betula verrucosa* (122 ± 28 grains m^–3^ year^–1^; 0.28%), *Robinia pseudoacacia* (119 ± 26 grains m^–3^ year^–1^; 0.27%), *Ligustrum lucidum* (102 ± 19 grains m^–3^ year^–1^; 0.23%), *Quercus robur* (71 ± 18 grains m^–3^ year^–1^; 0.16%), *Typha angustifolia* (56 ± 12 grains m^–3^ year^–1^; 0.13%), *Acacia dealbata* (48 ± 12 grains m^–3^ year^–1^; 0.11%), *Salix babylonica* (28 ± 11 grains m^–3^ year^–1^; 0.06%), *Jacaranda mimosifolia* (8 ± 5 grains m^–3^ year^–1^; 0.02%), *Tilia americana* (5 ± 4 grains m^–3^ year^–1^; 0.01%) and *Ailanthus altissima* (2 ± 2 grains m^–3^ year^–1^; 0.00%).

According to the records, each species begins pollinating at different times (Fig 4a–4i), and the most important contributors begin pollinating during the summer (Jan-Mar) and are *Crinodendron patagua* and then Palmae, displaying an airborne pollen maxima of approximately 30 and 10 grains m^–3^, respectively; they generate another less intense cycle during the spring (Oct-Dec) with a maximum of approximately 5 grains m^–3^. The species *Pinus spp*. presents a cycle in early autumn (Apr-May, maximum ≈10 grains m^–3^) and a second more intense cycle in winter (Aug-Sep) when it reaches a maximum of approximately 20 grains m^–3^. However, the species *F*. *excelsior* only presents an annual cycle with a maximum of approximately 50 grains m^–3^ observed at the end of autumn (May-Jun). *Cupressus sempervirens* has a lengthy pollination between July and October, *i*.*e*., during the winter and early spring, with a maximum of approximately 50 grains m^–3^. *Olea europaea*, *Acer negundo* and *Platanus orientalis* present a similar pollination period that is primarily between September and October, *i*.*e*., between the end of winter and early spring; however, they differ in their maximum by two orders of magnitude (40,200 and 2,000 grains m^–3^, respectively).

According to the allergenic potential [46], we can infer the possible health effects of pollens from different trees. Therefore, the pollens of trees classified as "severe allergens" that were observed in the study are the following: *Acer negundo*, *Cupressus sempervirens*, *Fraxinus excelsior* and *Olea europaea*, which are the five most abundant pollens of the trees in the study area and represent approximately 15% of the total pollens of the trees. *Platanus orientalis* (with an annual average pollen grain concentration of 31,951 ± 3,820 grains m^–3^ year^–1^) represents 72.0% of the tree pollen and is the most abundant pollen found in the study area. Other pollens classified as moderately allergenic were found in the study in a minor proportion, such as *Spanish avellano*, *Populus alba*, *Tilia americana*, *Ulmus pumila* and *Salix babylonica*. The total contribution of each of these pollens is less than 1.5% of the total tree pollens. Generally, the effect of tree pollen on the occurrence of pollinosis is important in different cities around the world [58–63].

### Weed pollen

**Fig 5:**
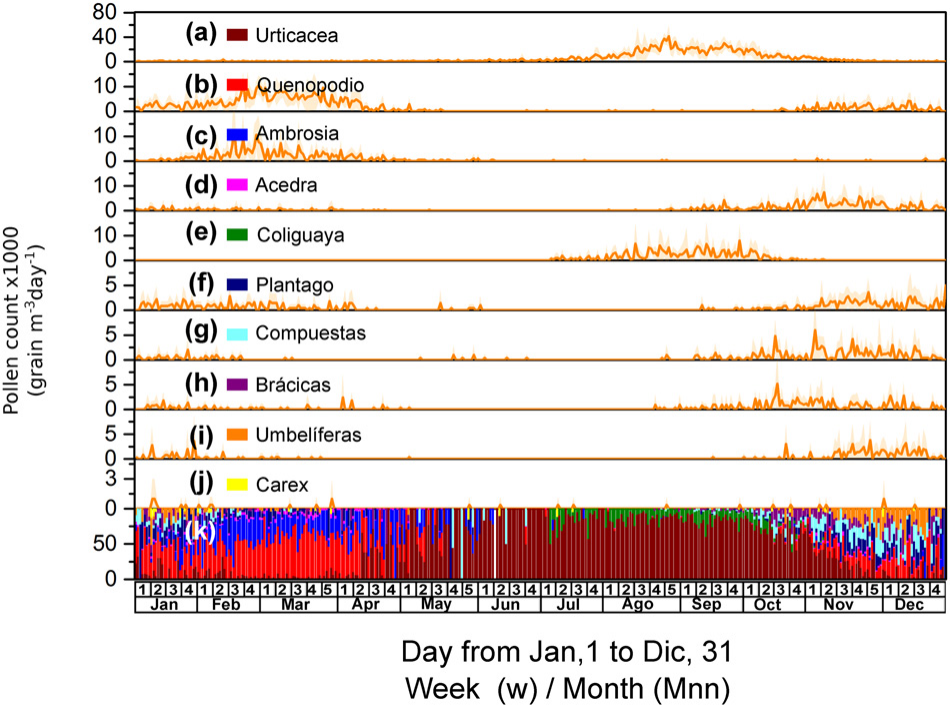
Average daily counts and relative contribution of the weed airborne pollen. (a to j) Average daily counts of the pollen grains from the most representative weed taxa and their corresponding standard deviations (expressed as grains m^–3^ day^–1^). (k) Relative contribution of each taxon to the daily counts of the weed pollen grains.

The ten different weed taxa that were identified in the pollen samples are shown in Fig 5a–5j. The contribution of each of these taxa to the average amount of the weed pollen grains collected per year over the study period is as follows: *Urticacea* (1,392 ± 104 grains m^–3^ year^–1^; 50.9%), *Chenopodium album* (567 ± 39 grains m^–3^ year^–1^; 14.9%), *Ambrosia elatior* (267 ± 39 grains m^–3^ year^–1^; 7.0%), *Rumex acetocela* (261 ± 28 grains m^–3^ year^–1^; 6.9%), *Colliguaja odorifera* (231 ± 32 grains m^–3^ year^–1^; 6.1%), *Plantago lanceolata* (175 ± 20 grains m^–3^ year^–1^; 4.6%), *Compositae spp*. (136 ± 20 grains m^–3^ year^–1^; 3.6%), *Brassica napus and Brassica campestris* (120 ± 18 grains m^–3^ year^–1^; 3.2%), *Umbeliferas spp*. (95 ± 19 grains m^–3^ year^–1^; 2.5%) and *Carex hirta* (12 ± 5 grains m^–3^ year^–1^; 0.3%). The corresponding daily relative contributions are presented in Fig 5k.

Pollination of the different weed taxa is similar to that of the tree taxa because they start at different times (Fig 5a–5j). *Carex hirta* is the taxon that shows the smallest contribution, and because its contribution is low, it is impossible to establish its preferential period of pollination compared with the other taxa. *Umbeliferas spp*., *Brassica napus*, *Compositae spp*., *Plantago lanceolata*, *Rumex acetocela* and *Chenopodium album* present two cycles of pollination, one cycle during the summer (Jan-Mar) and another more intense cycle during the spring (Oct-Dec). For *Umbeliferas spp*., *Brassica napus*, *Compositae spp*. and *Plantago lanceolata*, the corresponding maxima are approximately 5 grains m^–3^ each, whereas for *R*. *acetocela* and *C*. *album*, the corresponding maxima are approximately 10 grains m^–3^ each. *Colliguaja odorifera* and *Urticacea* have a preferential pollination period between the end of winter and early spring (Aug-Oct), with maxima of approximately 10 and 40 grains m^–3^, respectively.

Generally, weed pollens are associated with severe and moderate allergenicity. Severe allergenicity has been observed in the pollens of *Urticacea*, *Ambrosia elatior*, *Brassica napus and Brassica campestris*. Moderate allergenicity has been observed in the pollens *Chenopodium album*, *Rumex acetocela*, *Plantago lanceolata* and *Carex hirta*. However, these pollens with severe and moderate allergenicity were found at low proportions in the atmosphere, not exceeding 6% of the pollens measured in the study. Therefore, the effect of these pollens on the health of individuals can be generally estimated as low.

### Grasses pollen

In the case of grass pollen, only one taxon, *Gramineae*, was considered due to the difficult identification of the different taxa in the microscopic observation. The corresponding annual time-series is displayed in Fig 3e. The pollination period presents two maxima: one maximum during the summer (Jan-Mar) and another more intense cycle between the end of the winter and early spring (Aug-Dec); the maxima are approximately 15 and 45 grains m^–3^ for each peak, respectively.

Grass pollen is the major cause of pollinosis in many parts of the world. The concentration of airborne *grass* pollen in the atmosphere is frequently related to the degree of symptoms in pollinosis patients. Generally, grass pollens demonstrate a severe to moderate allergenicity and represent approximately 6% of the total pollens in this study.

### Long-term trends

The long-term estimates of the concentration trends were conducted according to the Theil-Sen estimator [53,54]. The OpenAir package in the programming language R was used to perform the estimates for the total amount of the collected airborne pollen, the airborne pollen from trees, grasses, weeds and indeterminate sources and the meteorological variables, such as the average daily temperature and the average daily relative humidity. The deseasonalized trends are summarized in Table 3.

**Table 3:** Summary of the time trend analysis using the deseasoned Theil—Sen method from 2009–2013 for the pollen concentrations. The table shows the median slope in % year^−1^.

*Station*	*Slope*, *unit year* ^*−1*^ *(% year* ^*−1*^ *)*	*95 CI unit year* ^*−1*^ *(% year* ^*−1*^ *)*	*p*
Total (grain m^-3^ day^-1^)	-1.65 (-1.19)	-3.85 (-2.68) - 0.98 (0.75)	> 0.1
Trees (grain m^-3^ day^-1^)	-0.03 (-0.02)	-0.95 (-0.81) - 1.28 (1.17)	> 0.1
Weeds (grain m^-3^ day^-1^)	-0.65 (-5.07)	-0.97 (-7.23) - -0.33 (-2.60)	< 0.001
Grass (grain m^-3^ day^-1^)	-0.36 (-3.52)	-0.61 (-5.59) - -0.16(-1.64)	< 0.001
Indet (grain m^-3^ day^-1^)	-0.16 (-4.45)	-0.29 (-7.29) - -0.03 (-0.99)	< 0.05

The 95% confidence interval of the slope and the *p* trend indicate statistically significant results.

The results reveal that for the airborne pollen from trees, the long-term concentration trend is not statistically significant (*p* > 0.1), and this result is verified when the confidence interval includes a slope of zero. The results for the airborne pollen from trees are similar to the total concentration of the airborne pollen, which has an observed trend of −1.65 units year^−1^ (–1.19% year^−1^), which is not statistically significant. This finding is most likely a result of the tree pollen representing more than 60%, on average, of the total amount of pollen. However, for weeds and grasses, a negative trend of—0.65 units year^−1^ (–5.07% year^−1^) and—0.36 units year^−1^ (–3.52% year^−1^), respectively, can be observed, with a confidence level of 99.9%. For the concentration of the airborne pollen from indeterminate sources, a negative trend is also observed at—0.16 units year^−1^ (–4.45% year^−1^); however, the associated confidence level is 95.0%. The trends observed for the concentrations of the airborne pollen from weeds, grasses and indeterminate sources are unclear and will be the subject of a future study.

### Meteorological variables

Table 4 shows the average meteorological variable temperature (T), the daily average relative humidity (RH), the daily predominant wind speed (ws) and wind direction (wd) and the results of the Theil-Sen (TS) estimator for the study period. An inspection of the results listed in Table 4 reveals that there are no major differences among the variables of T, RH, ws and wd over the years under study (see the maximum and minimum annual averages in Table 4). Furthermore, the ANOVA results show no statistically significant differences (*p* > 0.1) over the study years.

**Table 4:** Summary of the annual average (Avg), annual maximum (Max) and minimum (Min), with their respective standard deviations (SD) and the trend analysis using the deseasoned Theil—Sen method from 2009–2013 for the meteorological variables (temperature (T), daily average relative humidity (RH), daily predominant wind speed (ws) and wind direction (wd)).

*Station (unit)*	*Avg* ± SD	*Max* ± SD—*Min* ± SD	*Slope*, *unit year* ^*−1*^ *(% year* ^*−1*^ *)*	*95 CI unit year* ^*−1*^ *(% year* ^*−1*^ *)*	*p*
T(°C)	15.2 ± 5.5	16.3 ± 5.1 - 14.6 ± 5.5	-1.16 (-1.02)	-0.43 (-2.63) - 0.11 (0.71)	> 0.1
HR (%)	61 ± 15	61 ± 13 - 60 ± 15	0.05 (0.08)	-0.66 (-1.07) - 0.69 (1.21)	> 0.1
ws (ms-1)	0.26 ± 0.17	0.28 ± 0.17 - 0.24 ± 0.16	-0.01 (-4.17)	-0.03 (-8.54) - 0.01 (2.51)	> 0.1
wd (grad)	180 ± 104	190 ± 106 - 166 ± 103	-16.6 (-7.92)	-38.9 (-14.5) - 5.08 (3.44)	> 0.1

The table shows the median slope in % year^−1^. The 95% confidence interval of the slope and the *p* trend indicate statistically significant results.

However, the meteorological variables in this study (daily average T, RH, ws and wd) do not present statistically significant decreasing or increasing trends (*p* > 0.1); i.e., the confidence interval obtained from the TS estimator includes a slope of zero (Table 3). Accordingly, the variations in the long-term concentration trends of the airborne pollen from the weeds, grasses and indeterminate sources most likely would not be a result of the meteorological changes.

**Fig 6:**
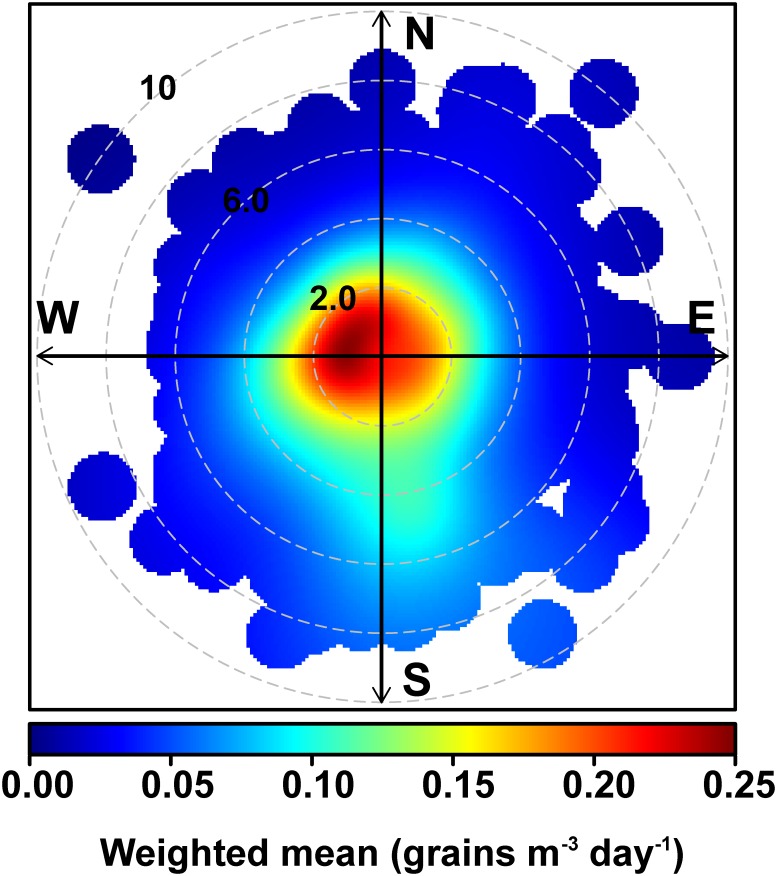
Bivariate polar plot of the weighted mean of the total pollen grain counts for the study site (concentric circles represent the wind speed in m s^–1^).

Fig 6 shows the bivariate polar plot of the weighted mean obtained using the polar plot option in the OpenAir package [53]. According to the results, the most significant contributions to the airborne pollen may be primarily of local origin because the greatest weighted means are observed at wind speeds of < 0.2 m s^–1^. However, a minor component originating from the SSE can be observed, and this component may be attributable to the wind pattern in the city.

### Comparison of the airborne pollen concentrations during 1993–1996 and 2009–2013

A comparison of the results obtained in the present study with those in the study by Rojas and Roure in 2001 [64], in which the authors have reported airborne pollens with concentrations higher than 1% of the total pollen grain counts using the same type of sampler used in this study, reveals significant differences between the airborne pollen loads from 1993 to 1996 and from 2009 to 2013 (Table 5).

**Table 5:** Summary of the total and average pollen grain counts for this study and for the study by Rojas and Roure (2001).

Pollen type or family	Rojas and Roure, 2001		This work	
	Year 1993–96		Year 2009–2013	
	Annual average 3-years	% On the total	Annual average 5-years	% On the total
*Platanus orientalis*	11,175	46.3	31,951	61.8
*Poaceae or Gramineae*	1,831	7.6	3,010	5.82
*Acer negundo*	1,715	7.1	3,394	6.60
*Cupressus sempervirens*	874	3.6	2,572	4.98
*Chenopodium album*	642	2.7	569	1.10
*Urticae*	583	2.4	1,932	3.74
*Fraxinus excelsior*	581	2.4	1,162	2.25
*Morus alba*	551	2.3	939	0.4
*Plantago lanceolata*	526	2.2	172	0.33
*Oleaceae Total*	676	2.1	^(^ ^a^ ^)^1,915	3.7
*Fabaceae Total*	413	1.7	^(^ ^b^ ^)^167	0.32
*Populus alba*	342	1.4	621	1.2
*Pinus spp*.	336	1.4	2,096	0.8
*Ulmus pumila*	323	1.3	1,341	0.5
*Palmae*	313	1.3	478	0.92
*Eucalyptus globulus*	292	1.2	988	0.4
*Brassica napus and campestris*	263	1.1	601	0.2
*Compositae spp*.	254	1.1	136	0.26
*Umbeliferas spp*.	246	1.0	96	0.19
*Indeterminate*	808	3.3	2,402	0.9
TOTAL	24,146	100	51,699	100

^(a)^ Sum of Olive, Ligustrum and Fresno;

^(b)^ sum of Aromo and Robinia

In the present study, the annual average of the total pollen grain counts (51,699 grains m^–3^ year^–1^ from 2009–2013) is 2.1 times higher than the corresponding count determined by Rojas and Roure [64] (24,146 grains m^–3^ year^–1^ from 1993 and 1996), although 103 types of pollen grains (compared with the 39 determined in the present study). To facilitate the comparison, the nomenclature of the study by Rojas and Roure is used in Table 5. In both of the studies, the primary pollen contributors are the same. In our study, these contributors are as follows (in decreasing order of importance): *Platanus orientalis*, *Acer negundo*, *Poaceae* or *Gramineae*, *Cupressus sempervirens*, *Chenopodium album* and *Urticacea*. The total amount of the contributors represents 81.4% of the total pollen grain counts. In the study by Rojas and Roure (2001), the same top six contributors are reported, which represent 69.7% of the total pollen grain counts; the only difference is that the order among *Acer negundo* and *Poaceae* or *Gramineae* and between *Chenopodium album* and *Urticacea* is inverted. However, the contribution of the major taxon increased by 2.9 times in more than a decade, from 11,175 to 31,951 grains m^–3^ year^–1^, and its contribution to the total pollen grain counts increased from 46.3% from 1994–1996 to 61.8% from 2009–2013. For *Poaceae* or *Gramineae* and *Acer negundo*, the concentration increase from 1994–1996 to 2009–2013 was 1.6 and 2.0 times, respectively. Notably, the increases observed for the average counts per year of the airborne pollen grains during the same period were *Cupressus sempervirens* (2.9 times), *Urticacea* (3.3 times), *Fraxinus excelsior* (2.0 times), *Pinus spp*. (6.2 times), *Ulmus pumila* (4.2 times) and *Eucalyptus globulus* (3.4 times). These differences may be explained as resulting from a process of urban expansion in the SChMA and possibly from an increase in the intensity of forestation [65].

Certain taxa, such as *Palmae*, *Compositae* and *Apiaceae*, presented a decrease in their average pollen grain counts per year from 1993 to 1996 [64] and from 2009 to 2013 (this study). However, these taxa represented exceptions to the trend of an increasing concentration demonstrated for the majority of the studied airborne pollen grains.

### Airborne pollen threshold levels

The counts of the pollen grains from trees, weeds and grasses were classified in accordance with the threshold levels of the airborne pollen concentration (grains m^–3^ day^–1^) provided in the recommendations of the AAAAI’s National Allergy Bureau (NAB).

The NAB threshold levels are as follows [66]. For tree pollen, a count is considered low between 1 and 14 grains m^–3^ day^–1^, moderate between 15 and 89 grains m^–3^ day^–1^, high between 90 and 499 grains m^–3^ day^–1^ and very high over 500 grains m^–3^ day^–1^. For grass pollen, the count is considered low between 1 and 4 grains m^–3^ day^–1^, moderate between 5 and 19 grains m^–3^ day^–1^, high between 20 and 199 grains m^–3^ day^–1^ and very high over 200 grains m^–3^ day^–1^. For weed pollen, the count is considered low between 1 and 9 grains m^–3^ day^–1^, moderate between 10 and 49 grains m^–3^ day^–1^, high between 50 and 499 grains m^–3^ day^–1^ and very high over 500 grains m^–3^ day^–1^.

**Fig 7:**
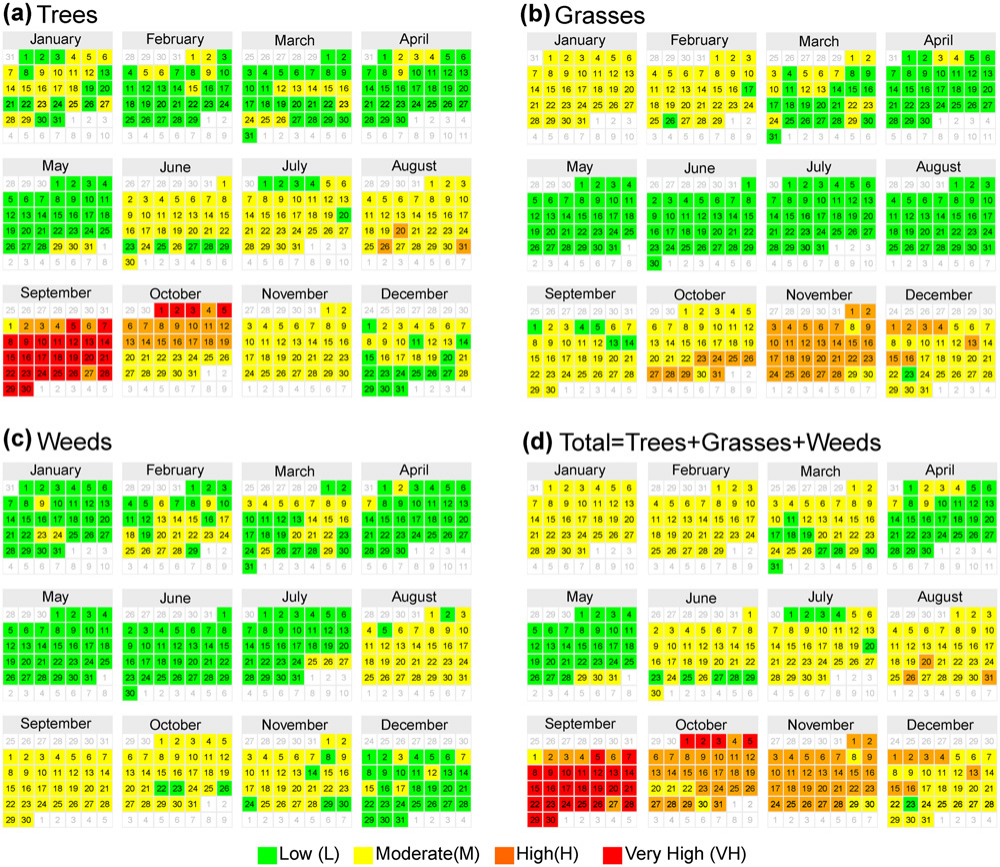
Calendar of risk levels of the average total airborne pollen concentration per day (estimated from the data collected daily from 2009 to 2014) for the groups: (a) trees, (b) grasses, and (c) weeds. (d) total pollen.

The results obtained relating the type of pollen grain to the corresponding threshold levels, which were previously indicated, are shown in Fig 7 as the average daily counts of the pollen grains. Thus, for tree pollen (Fig 7a), the average daily grain counts for 7.7% and 6.3% of the days per year correspond to the very high and high categories, respectively; 48.9% of the days per year correspond to the moderate category, and 36.9% of the days per year correspond to the low category. The grass pollen grain counts (Fig 7b) for 11.5% of the days per year correspond to the high category, and 40.2% and 48.4% of the days per year correspond to the moderate and low categories, respectively. Weed pollen grain counts (Fig 7) for 42.6% of the days per year correspond to the moderate category, and 57.4% of days per year correspond to the low category. Finally, combining the previous results (Fig 7d), at least 7.7% of the days per year are in the very high category, 17.8% are in the high category, 54.8% are in the moderate category, and only 19.7% are in the low category.

These results indicate that in the SChMA, the airborne pollen concentration is at high or very high levels during one-third of the days of the year, and these conditions may affect the health of people susceptible to respiratory and allergic diseases.

These results provide evidence of the large emission of pollens from trees. Therefore, it is possible to consider the allergenic potential of the pollen from trees and plants in future policies for planning and designing new urban spaces to give preference to species that release low amounts of pollen and whose pollen has a low sensitizing strength [67,68]. In addition, the convenience of gradual and rational replacement of the existing trees with trees and plants whose pollen, once released, is not allergenic must be considered. Similarly, certain management procedures (*e*.*g*., pruning or fumigation) in green areas must be conducted to facilitate the reduction of pollen release into the environment.

### Strengths and limitations of the study

The total green area coverage in the SChMA is 38.3 x 10^6^ m^2^, of which 62% is made up of a reduced number of green areas that are larger than one hectare [69]. The availability of green spaces in the SChMA is below the standard of 9 m^2^ ha^–1^ of green space per open dwelling recommended by the World Health Organization (WHO) and adopted by the publications of the United Nations Food and Agriculture Organization (FAO) [70]. In addition, according to the cadaster performed in 2003, the SChMA has an average of 3.4 m^2^ ha^–1^ [65]. The participation of each community in the total green area coverage demonstrates that of the 34 communities, only ten have more than 4% each. Of the remaining communities, seven have between 2% and 4%, and 17 have 2% [69].

Although the surface of the green areas is separated in the city, the type of vegetation is generally similar; thus, the types of pollen should also be similar. Therefore, the concentration that should vary as a function of the surface is observed in the green areas. In addition, the selected sampling site, the Providencia community, has a wide green coverage compared with other communities in the city, but one million people traverse the city daily, representing approximately 15% of the population of the city.

Considering the limitations of the study, the use of a certain number of locations for more intense pollen monitoring and the monitoring of meteorological variables (such as wind speed and direction) and atmospheric pollutants (such as particulate matter and gases), which may trigger or worsen the pollinosis, is recommended.

## Conclusions

The primary conclusions of the present study performed in the Santiago de Chile metropolitan area, in a sampling site located in Providencia County, are listed below.

- Tree pollen is the primary component of the airborne pollen collected and analyzed at the sampling site. The species *Platanus orientalis* (with pollen grains representing 61.8% of the total tree pollen grain counts) and *A*. *negundo* and *C*. *sempervirens* (with pollen grains representing 6.6% and 4.98%, respectively, of the total tree pollen grain counts) are the highest pollen contributors among the trees. Tree pollen is the major component of the pollen produced per year, possibly because of the growth of the city and the forestation of the green areas predominantly with *P*. *orientalis* and other exotic trees; these results may be extrapolated to other places in the city that have a similar proportion of green cover areas.
- Grass pollen (from Gramineae) is the third major component of the airborne pollen in the sampling area, contributing 5.82% of the total pollen grain counts. Among the weeds, the amount of airborne pollen collected from *Urticae* (3.74%) and *C*. *album* (1.10%) is remarkable.
- The airborne pollen concentrations reach a maximum in September, which coincides with a peak in the levels of tree pollen. Weeds reach their peak at the end of October, whereas grasses with pollen of a high allergenic strength have a corresponding peak in November. These results, because they depend on the physiology of the plants, may be extrapolated to all cities.
- A two-fold increase in the total amount of airborne pollen is observed from 1993–1996 to 2009–2013, which may have resulted from an increased incidence of pollinosis in the population. The pollen from *P*. *orientalis* shows a 2.9-fold increase during the same period.
- When the results obtained at the sampling site in SChMA are compared with the threshold levels for human health protection recommended by the American Academy of Allergy, Asthma and Immunology, 19.7% of the days in an average year are found in the low category, 54.8% are found in the moderate category, 17.8% are found in the high category, and 7.7% are found in the very high category. Thus, we concluded that the SChMA population is exposed to high and very high levels of allergens resulting from airborne pollen 93 days per year on average. It is necessary to consider that the coverage of green areas varies in different parts of the city; consequently, this result may differ from place to place.
- A recommendation based on the results of this study is to increase coverage of the aerobiological monitoring stations in the SChMA and in urban areas in which pollinosis is a relevant problem, to gather information that might help improve the quality of life of the inhabitants.
- The results of this study might be significantly useful for future epidemiological investigations that must consider the synergistic associations occurring between pollen and other atmospheric pollutants (such as particulate matter and gases) and possibly affecting the thresholds of human health resilience in the case of sensitive persons. The findings will be significantly useful for planning and managing the friendliest urban green zones for people with pollen sensitivities.

## Supporting Information

S1 Dataset Santiago Pollen 2009–2013 database. (XLS)
